# Biochemical and Functional Characterization of *Parawixia bistriata* Spider Venom with Potential Proteolytic and Larvicidal Activities

**DOI:** 10.1155/2014/950538

**Published:** 2014-05-07

**Authors:** Gizeli S. Gimenez, Antonio Coutinho-Neto, Anderson M. Kayano, Rodrigo Simões-Silva, Frances Trindade, Alexandre de Almeida e Silva, Silvana Marcussi, Saulo L. da Silva, Carla F. C. Fernandes, Juliana P. Zuliani, Leonardo A. Calderon, Andreimar M. Soares, Rodrigo G. Stábeli

**Affiliations:** ^1^Centro de Estudos de Biomoléculas Aplicadas a Saúde, CEBio, Fundação Oswaldo Cruz, Fiocruz Rondônia e Departamento de Medicina, Universidade Federal de Rondônia, UNIR, 76812-245 Porto Velho, RO, Brazil; ^2^Laboratório de Entomologia Médica, Fundação Oswaldo Cruz, Fiocruz Rondônia e Laboratório de Bioecologia de Insetos, Departamento de Biologia, UNIR, 76812-245 Porto Velho, RO, Brazil; ^3^Departamento de Química, Universidades Federal de Lavras, UFLA, 37200-000 Lavras, MG, Brazil; ^4^Departamento de Química, Biotecnologia e Engenharia de Bioprocessos, Universidade Federal de São João del Rei, UFSJ, Campus Altoparaopeba, 36420-000 Ouro Branco, MG, Brazil

## Abstract

Toxins purified from the venom of spiders have high potential to be studied pharmacologically and biochemically. These biomolecules may have biotechnological and therapeutic applications. This study aimed to evaluate the protein content of *Parawixia bistriata* venom and functionally characterize its proteins that have potential for biotechnological applications. The crude venom showed no phospholipase, hemorrhagic, or anti-Leishmania activities attesting to low genotoxicity and discrete antifungal activity for *C. albicans*. However the following activities were observed: anticoagulation, edema, myotoxicity and proteolysis on casein, azo-collagen, and fibrinogen. The chromatographic and electrophoretic profiles of the proteins revealed a predominance of acidic, neutral, and polar proteins, highlighting the presence of proteins with high molecular masses. Five fractions were collected using cation exchange chromatography, with the P4 fraction standing out as that of the highest purity. All fractions showed proteolytic activity. The crude venom and fractions P1, P2, and P3 showed larvicidal effects on *A. aegypti*. Fraction P4 showed the presence of a possible metalloprotease (60 kDa) that has high proteolytic activity on azo-collagen and was inhibited by EDTA. The results presented in this study demonstrate the presence of proteins in the venom of *P. bistriata* with potential for biotechnological applications.

## 1. Introduction

Animal venoms share common characteristics and typically are characterized by complex combinations of proteins and peptides with great structural diversity. Important biochemical, physiological, and pathological tools for the development of new drugs have arisen due to research on animal venoms [1, 2].

In this context, the toxins purified from the venom of spiders have a high potential for pharmacological and biochemical study and may have biomolecules with great therapeutic and biotechnological applicability [3]. However, spider venoms are even less studied compared to marine organisms, scorpions and snakes, since components of only 174 of the 43,244 species cataloged (approximately 0.4%) have been totally or partially characterized [4, 5].

Regardless of the genus studied, it has been observed that the usual spider venoms are complex mixtures of toxins which cause numerous neurological and biochemical changes in various animals, including mammals [8]. Note also that the biological activity of spider venom is a result of its major constituents: proteins, polypeptides, neurotoxic polyamines, biogenic amines, enzymes, nucleic acids, neurotransmitters, amino acids, and inorganic salts [9–12].

*Parawixia bistriata *(Araneidae) is a neotropical colonial spider. The spider is found in Central America, the Amazon, and in Northeast and Southeast regions of Brazil and is the only species of the Araneidae family that presents social behavior, communal refuge, and cooperative hunting. Described as harmless to man, many adults of this species form colonies in summer. There are on average about 168 individuals per colony, although the colony size varies depending on habitat type [13, 14].

The crude venom of* P. bistriata* and its chromatographic fractions have been characterized, with some components such as beta-carbolines, polyamines, and phosphatases already having been identified. Diverse biological and pharmacological activities were described, such as insecticidal, anxiolytic, and anticonvulsant activities, reversible inhibition of A and B type monoamine-oxidases, inhibition of GABA and glycine transporters, and increased glutamate uptake [15–22].

The aim of this study included biochemically and functionally characterizing the venom of* Parawixia bistriata* through proteomics and assays measuring enzymatic, pharmacological, biological and toxic activities, contributing to the advancement of toxinology and proteomics, as well as the direct or indirect development of products of medical or scientific interest.

## 2. Materials and Methods

### 2.1. Materials

We obtained crude venom from glands of over 1800* Parawixia bistriata* females that was collected in February 2012 in Ribeirão Preto, São Paulo. The glands were macerated with Milli-Q water in an ice bath and filtered through 0.45 micron filters. The filtrate was centrifuged (5 min at 2,500–5,000 ×g), and the supernatant was collected for lyophilization and maintained at −80°C. The study was authorized by CGEN/CNPq (010627/2011-1) and IBAMA (27131-2).

### 2.2. Animals

Male Swiss mice (18–20 g) were supplied by the vivarium of the Fiocruz Rondônia and received water and food ad libitum until the time of biological testing. The study was approved by the Committee of Ethics on the Use of Animals in Research (27131-1).

### 2.3. Functional Characterization

#### 2.3.1. Phospholipase Activity

The phospholipase A_2_ activity of the crude venom and the fractions was determined according to the protocol described by Holzer and Mackessy [23], modified for a 96-well plate. 4-Nitro-3-(octanoiloxi) benzoic acid (4N3OBA) was used as a substrate for reading the absorbance at a range of 425 nm, and the activity of phospholipase was determined in a directly proportional manner, establishing for each 0.10 AU (absorbance units) the presence of 25.8 nanomoles of chromophore (3-hydroxy-4-nitrobenzoic acid).

#### 2.3.2. Proteolytic Activity

Protease activity was determined in the presence and absence of 1 mM EDTA by hydrolysis of the azo-collagen colorimetric substrate (Sigma), according to the manufacturer's instructions [24], adapted to a 96-well plate, and an absorbance reading at 550 nm. An increase in absorbance of 0.05 was considered to be 1 unit of enzyme activity, and the result was expressed in units of enzyme activity per milligram of protein sample (unit/mg).

The proteolytic activity on fibrinogen was verified as described by Rodrigues et al. [25]. The hydrolysis of fibrinogen was demonstrated by SDS-PAGE using 12% polyacrylamide gels. To demonstrate the variability of enzymatic activity against different varying parameters, protein hydrolysis was observed after preincubation at different pHs (2.5 to 10.0), temperatures (−10°C to 100°C), and time intervals for enzymatic action (30 min to 24 h). Likewise, the effect of inhibitors or divalent ions was tested by incubating 20 *μ*g of venom with varying concentrations of heparin and EDTA (10, 20, and 30 mM) or ions (40 mM). The proteolytic activity on casein was tested as described by van der Walt and Joubert [26].

#### 2.3.3. Anti-Clotting

The clotting time in the presence of different concentrations of* P. bistriata* venom was evaluated with the addition of samples of venom 10 min before the CaCl_2_ (0.1 M) and time intervals after addition [27].

#### 2.3.4. Hemorrhagic Activity

To check the hemorrhagic activity of* Parawixia bistriata* venom, four groups of three male Swiss mice (28–32 g) were used. The samples with about 100 micrograms of total protein content were dissolved in 50 *μ*L of saline and intradermally inoculated in the dorsum of anesthetized mice with ethyl ether. Controls received 50 *μ*L of saline under identical conditions. After three hours, the animals were euthanized by cervical dislocation and the skin was removed, showing the activity based on the presence of a hemorrhagic halo [28].

#### 2.3.5. Edematogenic Activity

Groups of six male Swiss mice (18–22 g) were injected in the subplantar region, using from 10 to 200 *μ*g/animal of* P. bistriata* crude venom diluted in 50 *μ*L of PBS, and the negative control was injected only with PBS. After 0.5, 1, and 3 hours, edema in each paw was measured with the aid of a low-pressure spring gauge (Mitutoyo, Japan) [29]. The values taken at the beginning were subtracted and the difference was reported (mean ± standard deviation).

#### 2.3.6. Lethality

Lethality induced by* Parawixia bistriata *venom was evaluated by intraperitoneal injections of samples (100, 250, and 500 *μ*g) in groups of mice of 18–22 g (sample size = 6, within 48 hours). 100 *μ*L PBS was used as a negative control and snake venoms that are known to induce lethality in less than 5 h at these concentrations were used as positive controls [30].

#### 2.3.7. Myotoxicity

Myotoxic activity was assayed by measuring the release of creatine kinase (CK) as recommended by Stábeli et al. [31] using a kinetic CK-UV Kit (Bioclin, Brazil). Solutions of 10 to 200 *μ*g/animal of* P. bistriata *venom diluted in 50 *μ*L of phosphate buffered saline (PBS) were applied intramuscularly in six male Swiss mice weighing 18–22 g. For negative controls, we used the same amount of 0.15 M phosphate buffered saline. After 3 hours, blood was collected from the tails of the mice into heparinized tubes and then centrifuged to separate the plasma. The latter was incubated for 3 min at 37°C with 1.0 mL of CK-UV and the amount of CK was determined according to the phosphorylation of a *μ*mol of creatine/min at 25°C.

#### 2.3.8. Genotoxicity

Genotoxicity was observed using two methods: a micronucleus test and a comet assay. The experiments were approved by the Ethics Committee of FCFRP-USP (102/2009) and the results were analyzed statistically and expressed as mean ± standard deviation (SD) (Kruskal-Wallis).

The micronucleus test in human lymphocytes* in vitro* was performed according to the technique of Moorhead et al. [32] with modifications described by Marcussi et al. [33].* Parawixia bistriata* venom was added to the cells at concentrations of 2.5, 5, 15, and 30 *μ*g/mL at 24 h after the start of the cultures. The antineoplastic cisplatin 6 *μ*g/mL (PLATINIL, Chiral Chemicals of Brazil SA) was used as a positive control. The criteria used to evaluate the micronuclei were previously described by Fenech [34].

For the comet assay we used the same methodology as Singh et al. [35]. Cell suspension of 10^6^ cells/mL was used to evaluate 100 nucleoides per slide in triplicate. The results were expressed in classes and arbitrary units as described by Collins et al. [7, 36], with the ranking in categories being adapted considering suitability for visual analysis and the levels of damage as described by Marcussi et al. [33].

#### 2.3.9. Larvicidal Activity

The larvicidal activity of* P. bistriata *venom and fractions was ascertained at concentrations from 5 to 15 ppm, using 1% ethanol as a negative control and performing the tests in quadruplicate for each concentration of the sample. Lethal concentrations (LCs) were calculated from 24 to 48 hours using Probit analysis and Weibull distribution (Minitab, Minitab Inc.). The samples were solubilized in water and transferred to 50 mL plastic cups where 25 3rd-4th instar* Aedes aegypti* larvae were added in standard laboratory conditions (27°C, 70% relative humidity, and 12 hours of photoperiod).

The tests were conducted in triplicate at different times and with different groups of larvae. Dying or debilitated larvae were counted as dead, according to the criteria established by the World Health Organization and similar to that performed by Furtado et al. [37]. RM Two-Way Anova (Prism 6-GraphPad) was used to analyze the effect of venom and fractions at different times and concentrations on larval mortality.

#### 2.3.10. Antibacterial and Antifungal Activity

To test the sensitivity of dermatophytes and yeast, we followed protocol M-38-P, described by the National Committee for Clinical and Laboratory Standards [38]. The minimal inhibitory concentration (MIC) was determined by visual reading of the development of fungal strains of* Trichophyton rubrum *(ATCC MYA-3108) and* Candida albicans* (ATCC 10231). To evaluate the sensitivity of the bacteria* Staphylococcus aureus* (ATCC 6538),* S. epidermidis* (ATCC 2228),* Escherichia coli* (ATCC 25922), and* Pseudomonas aeruginosa* (USP) against crude venom, we used microdilution plates described by protocol M2-A8 of the National Committee for Clinical and Laboratory Standards [39], which was used in similar experiments described by Stábeli et al. [40].

### 2.4. Biochemical Characterization

#### 2.4.1. Protein Content

The dosage of crude venom proteins was performed using the reagent Bradford [41] using a standard curve obtained with bovine serum albumin concentrations (BSA) with *R* ^2^ ≥ 0.99. The absorbance was read using 96-well plates (Biotek) at 595 nm.

#### 2.4.2. Chromatography

The purification of proteins was carried out using cation exchange chromatography on a CM Hytrap column and reverse phase chromatography on a C18 column. Thus, there were two chromatographic steps: first, cation exchange chromatography on a 1 mL CM FF Hytrap column followed by reverse phase HPLC on a Discovery C18 column (25 × 4.6 mm Supelco), both of which were done on an Akta purifier chromatograph-GE. In the cationic exchange chromatography, 10 mg of* Parawixia bistriata* crude venom was diluted in 1 mL of 20 mM ammonium bicarbonate and centrifuged for 5 min at 13,000 ×g and applied to the column. Elution was performed with 20 mM ammonium bicarbonate (eluent A) and 500 mM ammonium bicarbonate (eluent B) at a gradient of 0 to 100% of eluent B and a flow rate of 1 mL/minute.

Then, the fractions were applied to reverse phase chromatography, diluted in 1 mL of 0.1% trifluoroacetic acid (TF), and centrifuged for 5 min at 13,000 ×g 0.1% TFA (eluent A) and a solution of 0.1% TFA and 99.9% acetonitrile (eluent B) was used at a gradient of 0 to 100% B at a flow rate of 1 mL/minute. The peaks were monitored at 280 nm absorbance, read in Software Dataq (Dataq, Inc.) collected manually, identified, lyophilized, and stored at −20°C.

#### 2.4.3. SDS-PAGE

SDS-PAGE was performed as described by Laemmli [42] with modifications. Approximately 2 *μ*L of protein/peptide (200 mg) from the venom of* Parawixia bistriata* was applied to discontinuous gel with dimensions of 180 × 160 × 1.0 mm. After the protein separation, the gel was stained using Coomassie Blue G-250.

#### 2.4.4. Two-Dimensional Electrophoresis

A sample of crude venom of* Parawixia bistriata* applied in two-dimensional electrophoresis was subjected to prior purification using* Clean-up Kit* (Sigma) according to the protocol of the manufacturer. The kit* deglycosylation enzyme mix-P6039S* (BioLabs) was used according to the manufacturer's instructions to study the deglycosylation of* Parawixia bistriata* crude venom. Two-dimensional electrophoresis was performed according to the method of O'Farrell [43] with modifications. During the focusing process, the proteins and peptides from the sample of* Parawixia bistriata *crude venom were separated based on isoelectric point in 13 cm strips of a polyacrylamide gel with pH values ranging from 3 to 10 nonlinearly.

After rehydration of the strips according to manufacturer's instructions, they underwent focusing, followed by reduction and alkylation according to the method of Vesterberg [44]. Then the strips were applied to 12.5% polyacrylamide gels for an electrophoretic run at 25 mA per gel and 100 W. The presence of proteins in the gel was revealed using colloidal Coomassie Blue G-250.

## 3. Results and Discussion

The biochemical, proteomic, and functional characterization of* Parawixia bistriata* venom presented in this study confirms the presence of various nonprotein toxins of low molecular weight previously described in advanced neurochemical and pharmacological research [15–22], revealing novel enzymatic and biological activities, as well as enzymatically active and inactive protein components of high molecular weight not characterized in previous studies.

Considering the predominant protein composition (670 *μ*g protein/mg of crude venom) in the* P. bistriata *venom, we chose to investigate the probable enzymatic activities of these proteins, testing mainly for phospholipase A_2_ and proteolytic activity with a number of different specific substrates. The phospholipase activity of the crude venom of* P. bistriata* was not significant against the substrate for PLA_2_, demonstrating low enzymatic activity when compared to the enzymatically active phospholipases from snake venom (data not shown). However, proteolytic activity can be considered the main enzymatic activity identified in the venom of* P. bistriata *in this study, since it tested positive for several different protein substrates, which are presented below.

**Figure 1:**
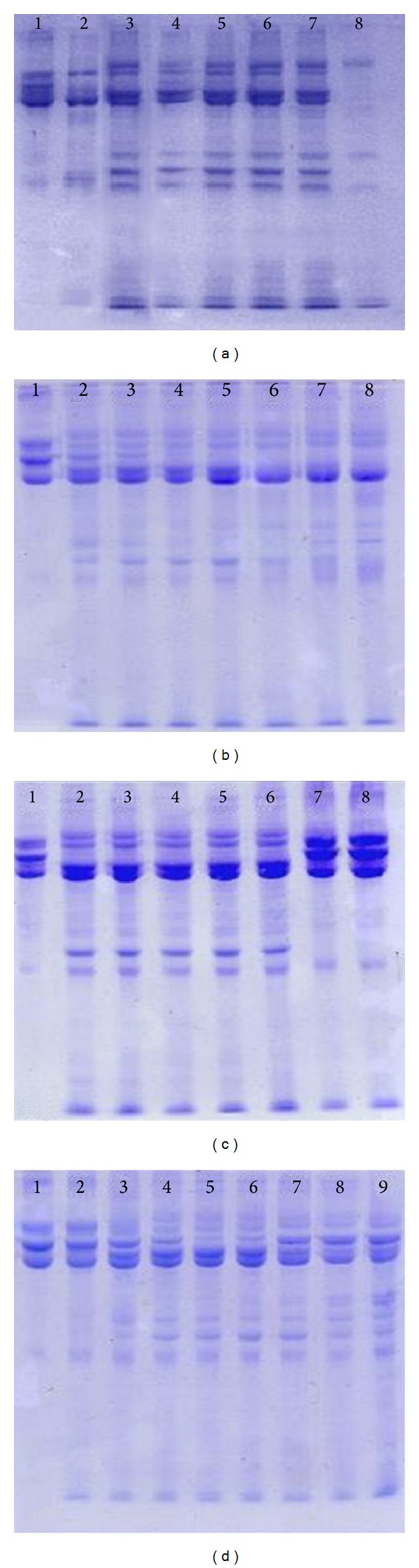
Proteolytic activity of* Parawixia bistriata* crude venom on Fibrogen. Fibrinogen was preincubated with the crude venom of* P. bistriata *in varying concentrations, times, temperatures, and pHs. The samples were subjected to SDS-PAGE for visualization of protein bands. Staining: Coomassie Blue.*F = Fibrogen **CV =* P. bistriata *crude venom. (a) Effect of concentration. Lanes: (a)1: *F (80 *μ*g); (a)2: F + **CV (10 *μ*g); (a)3: F + CV (20 *μ*g); (a)4: F + CV (40 *μ*g); (a)5: F + CV (60 *μ*g); (a)6: F + CV (80 *μ*g); (a)7: F + CV (100 *μ*g); (a)8: CV (50 *μ*g). (b) Effect of incubation time. Lanes: (b)1: F (80 *μ*g); (b)2: F + 20 *μ*g CV (30′); (b)3: F + 20 *μ*g CV (1 h); (b)4: F + CV (2 h); (b)5: F + CV (3 h); (b)6: F + CV (6 h); (b)7: F + CV (12 h); (b)8: F + CV (24 h). (c) Effects of different temperatures. Samples: (c)1: F (80 *μ*g); (c)2: F + CV (20 *μ*g) (−10°C); (c)3: F + CV (4°C); (c)4: F + CV (25°C); (c)5: F + CV (37°C); (c)6: F + CV (50°C); (c)7: F + CV (70°C); (c)8: F + CV (100°C). (d) Effects of different pHs. Samples: (d)1: F (80 *μ*g); (d)2: F + CV (20 *μ*g) (pH 2.5); (d)3: F + CV (pH 3.5); (d)4: F + CV (pH 4.5); (d)5: F + CV (pH 5.5); (d)6: F + CV (pH 7.0); (d)7: F + CV (pH 8.0); (d)8: F + CV (pH 9.0); (d)9: F + CV (pH 10.0).

**Figure 2:**
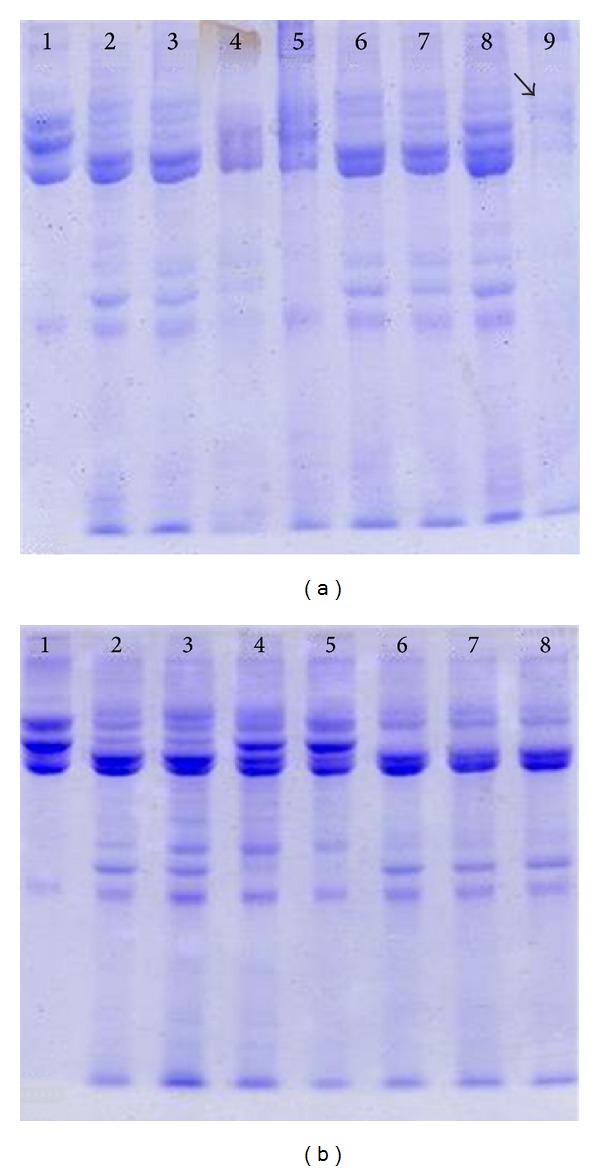
Fibrinogenolytic activity of* Parawixia bistriata* venom. Fibrinogen was preincubated with the crude venom of* P. bistriata* in the presence of divalent ions, EDTA and heparin. The samples were subjected to SDS-PAGE for visualization of protein bands. *F = Fibrinogen **CV =* P. bistriata *crude venom. (a) Effect of divalent ions: lanes: (a)1: *F (80 *μ*g); (a)2: F + **CV (20 *μ*g); (a)3: F + CV + Ca^2+^ (40 mM); (a)4: F + CV + Co^2+^ (40 mM); (a)5: F + CV + Fe^2+^ (40 mM); (a)6: F + CV + Mg^2+^ (40 mM); (a)7: F + CV + Mn^2+^ (40 mM); (a)8: F + CV + Na^+^ (40 mM); (a)9: F + CV + Zn^2+^ (40 mM). (b) Effect of EDTA and heparin. Lanes: (b)1: F (80 *μ*g); (b)2: F + CV (20 *μ*g); (b)3: F + CV + 10 mM EDTA; (b)4: F + CV + 20 mM EDTA; (b)5: F + CV + 30 mM EDTA; (b)6: F + CV + 10 mM heparin; (b)7: F + CV + 20 mM heparin; (b)8: F + CV + 30 mM heparin.

Proteolytic activity against fibrinogen is present in the crude venom at intensities that vary nonlinearly and are concentration-dependent. It was observed that the enzymatic activity of* P. bistriata *venom proteins oscillates when the sample (fibrinogen + crude venom) is subjected to varying parameters such as temperature, pH, and the presence of divalent ions and EDTA (Figures 1 and 2).

The proteolytic activity of the venom of* P. bistriata* on fibrinogen was concentration dependent and occurs by breaking the *α* and/or *β* chains and releasing fibrinopeptides. The enzymatic activity on fibrinogen was completely inhibited after preincubation at temperatures above 70°C (Figure 1) and when subjected to a solution with pH ≤ 2.5. After incubation in solutions of pH 3.5, 8.0, 9.0, and 10.0, there was a partial loss of fibrinogenolytic activity, similar to that observed after preincubation of the venom in the presence of Na^+^ ions or EDTA at concentrations of 20 and 30 mM. The Zn^++^ ion appears to potentiate the proteolytic effect of the venom by causing total breakage of the *α* and *β* chains and partial breakage of the *γ* chain (black arrow in Figure 2). Heparin showed no effect on this activity (Figure 2).

Besides its enzymatic activities, the venom of* P. bistriata* was also investigated for its functional biological activities. The venom of* P. bistriata* was not able to coagulate plasma from mice in the observation period of up to one minute at all concentrations tested. Nevertheless, it was noted that the venom of* P. bistriata* interferes with the clotting process (Table 1), by delaying the time of clot formation with all quantities of crude venom tested. It was observed that the lowest tested amount (10 *μ*g) was able to increase the coagulation time by approximately 30%; also observed were increased coagulation times by 86%, 160%, and 556% for doses of 25 *μ*g, 50 *μ*g, and 100 *μ*g, respectively. It was also noted that there was no coagulation of the plasma after 48 hours of observation in studies in which higher amounts of venom (200 and 250 *μ*g) were used.

Also, regarding interference from crude venom proteins in hemostasis, the venom of* P. bistriata *showed no hemorrhagic activity, since no hemorrhagic halo was observed after application of the venom in the test performed with mice (data not shown), in contrast to the hemorrhagic halo obtained in the positive control using* B. mattogrossensis *snake venom.

**Figure 3:**
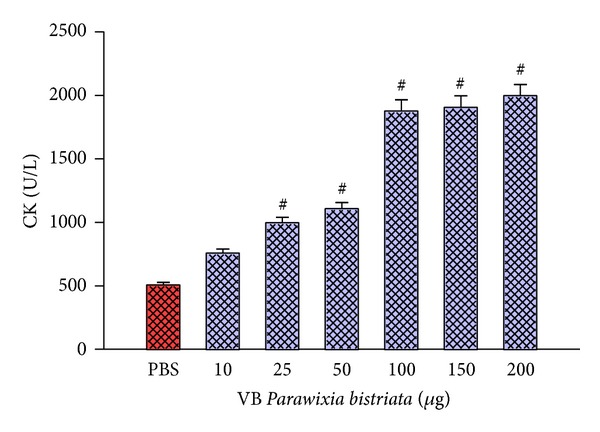
Myotoxic activity of* Parawixia bistriata *venom. Solutions from 10 to 200 *μ*g/animal of* P. bistriata* venom diluted in 50 *μ*L of PBS were applied intramuscularly in mice. The plasma was incubated for 3 min at 37°C with 1.0 mL of the reagent CK-UV. The amount of CK expressed after the phosphorylation of one *μ*mol of creatine/min at 25°C. Negative control: 0.15 M PBS. Test conducted using a kinetic CK-UV Kit (Bioclin, Brazil). ^#^Significantly different from the negative control (*P* < 0.05).

The venom of* P. bistriata* was also able to cause myotoxicity measured by the release of creatine kinase (CK) after intramuscular injection of venom in mice (Figure 3). Myotoxic activity of the venom of* P. bistriata* was significant starting from a dose of 25 *μ*g/animal, showing an increase of approximately 100% CK compared to PBS, the negative control. A dose of 200 *μ*g/animal increased the myotoxicity by 400% compared to that caused by PBS.

Although it triggers specific physiological responses, it was observed that the venom of* P. bistriata* is not lethal when injected intraperitoneally in mice at concentrations between 25 and 1,500 *μ*g/animal.

We also researched genotoxic activity in order to toxicologically characterize* P. bistriata* crude venom using two methods: the formation of micronuclei and a Comet test, which allowed for the analysis of crude venom induction of chromosomal damage or breakage of DNA strands.

The micronucleus test showed a greater percentage of mononuclear cells after treatment with the venom at all concentrations when compared to the percentage obtained for cisplatin. We also observed a lower percentage of tri or multinucleated cells compared to the same positive control, pointing out the low capacity of the venom to alter cell division (Table 2).

**Figure 4:**
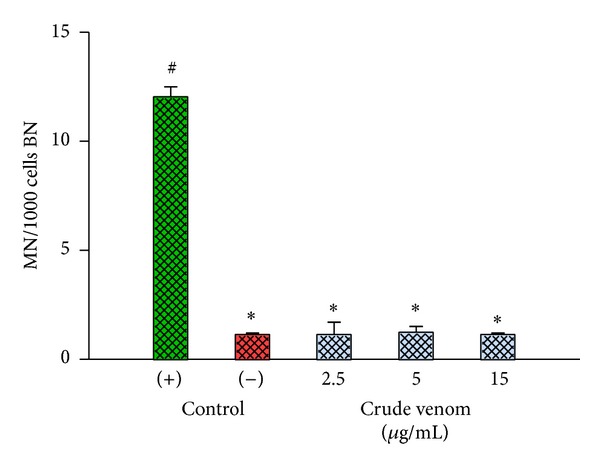
Distribution of micronuclei in binucleated human lymphocytes. Cells were treated with the venom of* P. bistriata* at concentrations of 2.5, 5, and 15 *μ*g/mL. The drug cisplatin (6 *μ*g/mL) was used as the positive control and the negative control was untreated cell cultures. The results are presented as mean ± SD (*n* = 4) of two individual experiments. *Statistically different from the positive control (*P* < 0.05). ^#^Statistically different from the negative control (*P* < 0.05).

It also showed less micronucleus formation in binucleate cells (MN/BN) after treatment with the venom of* P. bistriata*, indicating low induction of coarse damage to chromosomes (Figure 4).

**Figure 5:**
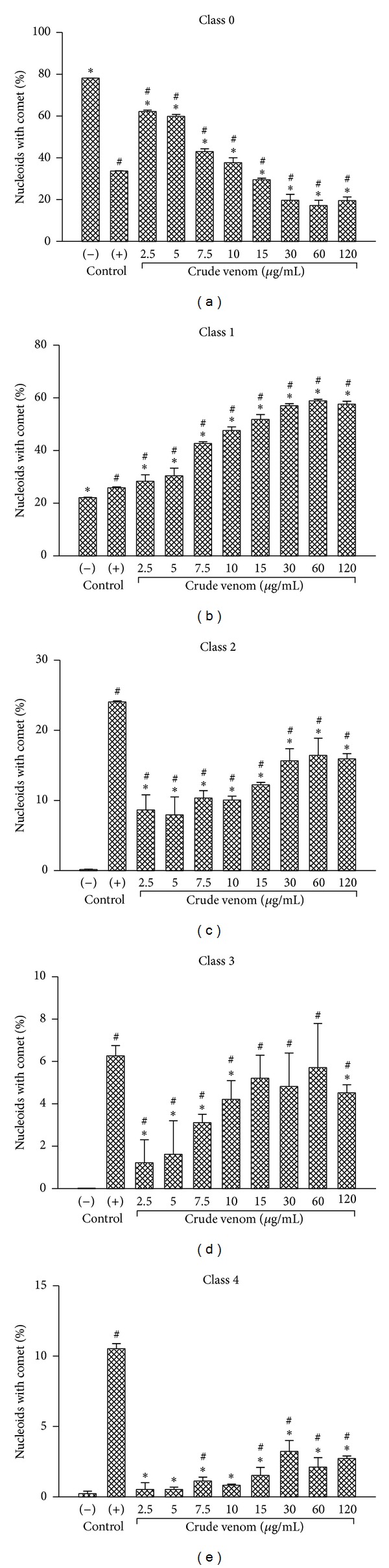
Frequency of nucleoids distributed in damage classes of Collins. The levels of damage to cells treated with crude venom of* Parawixia bistriata* were distributed in class 0 (no damage <5%), class 1 (low damage: 5 to 20%), class 2 (medium damage: 20 to 40%), class 3 (high damage: 40–85%), and class 4 (totally damaged >85%). Positive Control: doxorubicin (6 *μ*g/mL). Negative control: untreated cell cultures. *Statistically different from the positive control (*P* < 0.05). ^#^Significantly different from the negative control (*P* < 0.05).

In the comet test, there was a prevalence of classes 0, 1, and 2 from Collins [7, 36], with most of the nucleoids having less than 40% damage (Figure 5).

The comet test also showed a higher significant percentage of damage in nucleoids treated with* P. bistriata *venom, at concentrations of 7.5 *μ*g/mL and higher, compared to the positive control doxorubicin. It was found that the number of damaged nucleoids increased as the concentration of the sample increased in most of the tests (Table 3); however, global indices of damage proved to be independent of concentration or linear relationship meaning cytotoxicity should be considered.

The cytogenetic toxicological analysis of animal venoms and their isolated proteins is also of great importance for the identification and characterization of potential therapeutic agents, as well as for a better understanding of the mechanisms of action of these toxins in the human body [33]. The breakage of DNA can result in permanent damage, altering the morphology and physiological homeostasis of cells [34, 45]. Molecules from venoms capable of inducing genotoxicity may also participate in mutagenic and carcinogenic events, according to the biochemical characteristics of each individual.

Some studies have associated morphological and physiological changes in victims of accidents with venomous animals, especially for late sequelae, which could be a result of the cumulative effects of the toxins, resulting in neurodegenerative diseases and chronic inflammatory diseases [46, 47].

Besides the functional biological activities of the venom of* P. bistriata, *we also investigated the action of the constituents of the crude venom on microorganisms by tracking cytotoxic activity against fungi and gram positive and negative bacteria. The test results of the minimum inhibitory concentration did not show good antimicrobial activity against gram positive and negative; however, a concentration of 1 mg/mL (MIC) of venom completely inhibited macroscopic growth of* Candida albicans*.

Most antimicrobial toxins from previously described animal venoms are peptides [48–50]. The crude venom of* Parawixia bistriata *was active against* Candida albicans*, and the identification and isolation of the toxin responsible for this effect could reveal a new potential antifungal compound.

**Figure 6:**
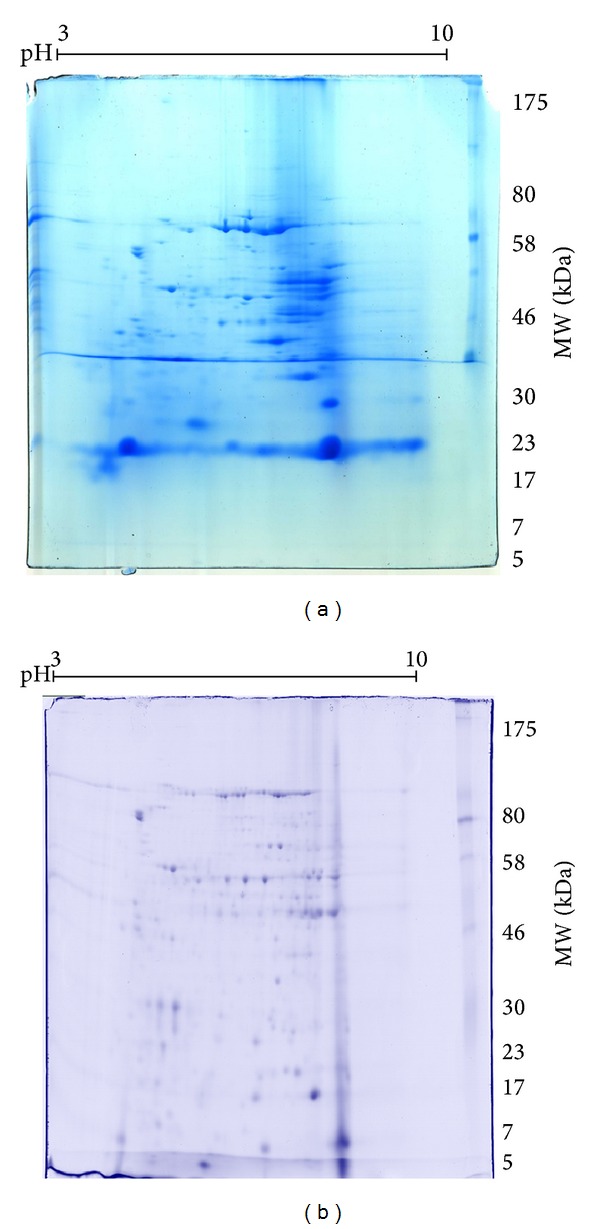
2D electrophoresis of* Parawixia bistriata* venom. Samples containing 2 mg of crude venom and 2 mg of deglycosylated venom underwent focusing and were then applied to 12.5% acrylamide gels-Item 3.5.4. Methods. Staining performed by colloidal Coomassie. (a) Two-dimensional gel electrophoresis of the crude venom of* P. bistriata*. (b) Two-dimensional gel electrophoresis of deglycosylated venom of* P. bistriata*. MW = molecular weight 7–175 kDa.

The venom proteins of* P. bistriata* were also characterized by 2D electrophoresis, with and without deglycosylation (Figure 6).

The crude venom revealed several bands corresponding to proteins of high molecular weight, ranging mostly from acidic to neutral pH. The deglycosylated venom demonstrated a different migration profile of protein bands, especially for proteins with a low molecular weight, for gels with the same concentration of acrylamide that were run under the same experimental conditions.

Two-dimensional electrophoresis of* P. bistriata *venom showed various proteins of high molecular weight (mostly above 40 kDa), which could be identified as evidence of the presence of several enzymes [51] previously described in the* Loxosceles intermedia *spider [52].

Glycosylated proteins are common in animal [53, 54]. Some components of snake venom are known to possess glycosylation; however, little is known about the structure of the carbohydrates present in these proteins [55].

Through the combined analysis of chromatographic profiles from cation exchange chromatography followed by reverse phase chromatography, the predominance of polar proteins with acidic or neutral pHs was observed in the crude venom.

**Figure 7:**
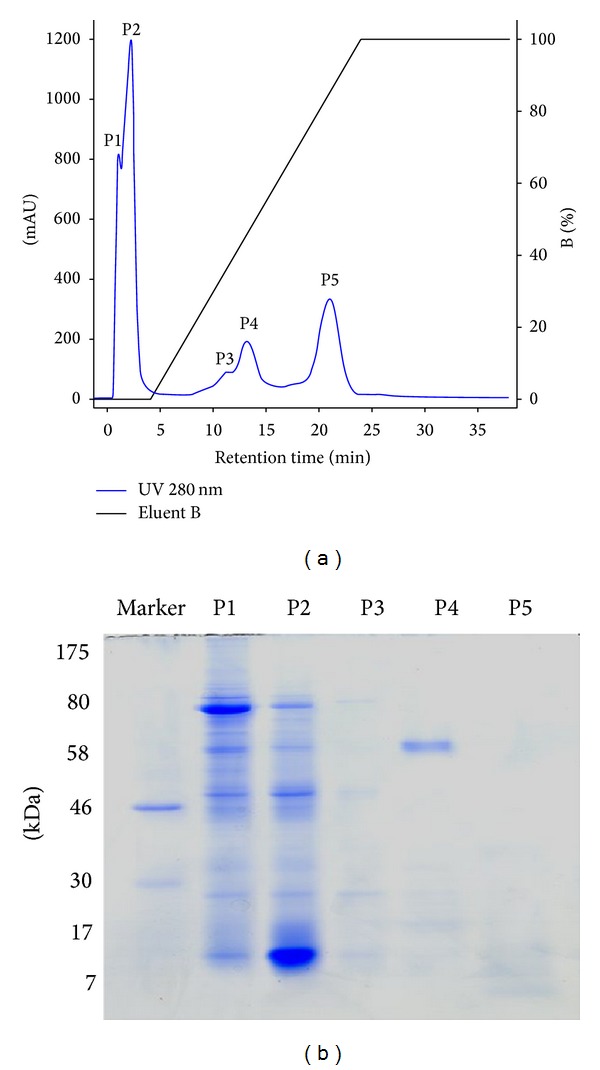
Chromatographic and electrophoretic profile of* Parawixia bistriata* crude venom. (a) Cation exchange chromatography of 2.7 mg of the crude venom of* Parawixia bistriata*. A 1 mL FF-CM Hytrap (7 × 25 mm) column was used at a flow rate of 0.5 mL/min. Gradient of 0 to 100% of eluent B. Eluent A: 20 mM ammonium bicarbonate, pH 8.0; eluent B: 500 mM ammonium bicarbonate, pH 8.0. Monitoring at 280 nm. (b) SDS-PAGE electrophoresis on 12.5% acrylamide gel of lyophilized fractions from cation exchange chromatography of* Parawixia bistriata* venom. Coomassie Blue staining. Molecular weight marker: 7–175 kDa.

Five fractions, named P1, P2, P3, P4, and P5, were collected during cation exchange chromatography (Figure 7(a)). The SDS-page of the fractions showed that most of the protein was concentrated in fractions P1 and P2, requiring the establishment of other purification strategies for the isolation of the proteins within these fractions to be considered in future studies. As shown in the electrophoresis, fraction P4 contained a protein of about 60 kDa with a significant degree of purity (Figure 7(b)). The electrophoresis showed protein constituents of high molecular weights in the range of 60 to 80 kDa but also showed the presence of constituents of about 14 kDa.

Considering the prior finding using SDS-page of an apparent molecular mass of approximately 60 kDa in the major protein fraction P4, we proceeded to compare possible enzymatic activities of all the fractions of* P. bistriata*, including fraction P4, with primary focus on the research of proteolytic enzyme activity in azo-collagen substrate and in the presence of the inhibitor EDTA.

**Figure 8:**
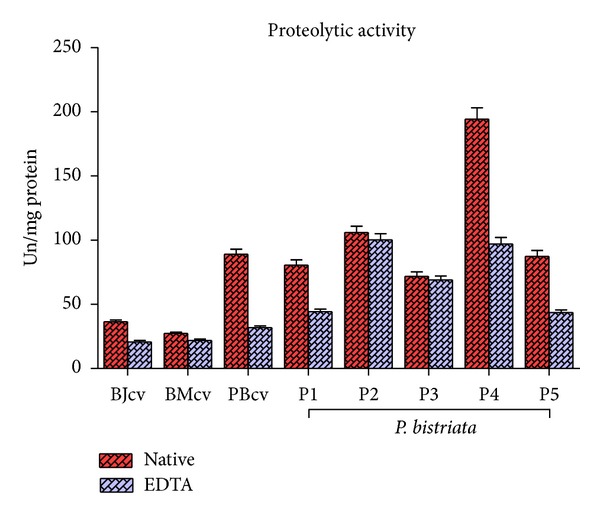
Proteolytic activity of* Parawixia bistriata* crude venom against the substrate azo-collagen. An increase in absorbance of 0.05 was regarded as 1 unit of enzymatic activity for each milligram of protein. The red bars represent the activity of the native protein and the light gray bars represent the activity of the proteins after incubation with 1 mM EDTA. BJcv:* B. jararacussu* crude venom; BMcv:* B. matogrossensis *crude venom. PBcv:* P. bistriata* crude venom. P1, P2, P3, P4, and P5: fractions from the venom of* P. bistriata*. Absorbance read at 550 nm.

Proteolytic activity was confirmed for the crude venom and all fractions (P1 to P5) for the substrate azo-collagen. The enzymatic activity of fractions P1, P4, and P5 was significantly reduced in the presence of 1 mM EDTA, suggesting that the enzymatic activity is dependent on metals (Figure 8). It was observed that the enzymatic activity of P1 was reduced by 45%, P4 reduced by about 50%, P5 reduced by about 50%, and the crude venom reduced by about 64% in the presence of 1 mM EDTA. No significant reduction in enzymatic activity was seen for fractions P2 and P3. Confirmation of proteolytic activity present in* P. bistriata *crude venom and its fractions points to the presence of proteases, some of which are inhibited by metal chelators. The P4 fraction contains the protease with greatest proteolytic activity and the highest degree of purification measured in this study, and we suggest that it belongs to the same class of metalloproteases with molecular weight in the range of 40–60 kDa. Proteases are present in the digestive fluids of different species of spiders, just as protease inhibitors have been reported and characterized in several studies [56–59].

Proteolytic enzymes can interfere with the blood coagulation cascade and induce hemorrhaging, edema, myonecrosis, dermonecrosis, and inhibition of platelet aggregation [60–63]; however, the crude venom of* P. bistriata* showed edematogenic and anticoagulant but no hemorrhagic activity.

Among the known biological activities present in animal venoms, insecticidal/larvicidal activity has been described in venoms of many species of spiders, so we sought to investigate the larvicidal activity of the crude venom as well as of the fractions obtained from the venom of* P. bistriata*. Larvicidal activity was found in all samples (Table 4) tested (crude venom and fractions P1, P2 and P3). Fractions P4 and P5 were not tested because of the limited amount of sample available for testing.

The lethal concentrations for* A. aegypti* were similar among the crude venom and fractions, but LCs values were much lower compared to larvicidal tests against this mosquito species using plant extracts and fractions by our group [64].

**Figure 9:**
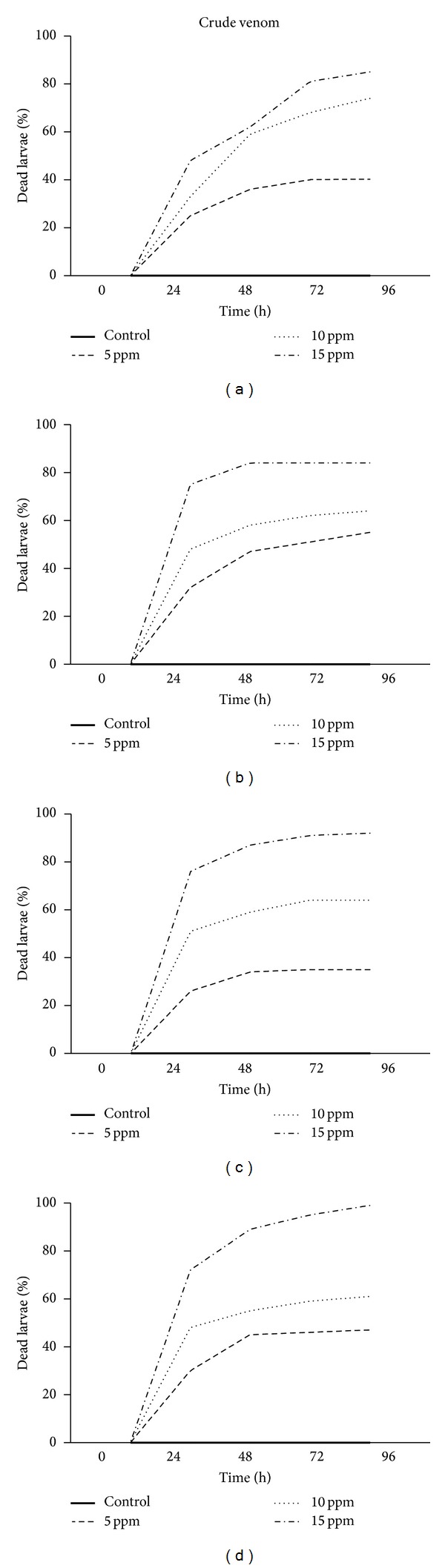
Larvicidal activity of the crude venom and chromatography fractions of* Parawixia bistriata* on* Aedes aegypti*. Concentrations of 5 to 15 *μ*g/mL of the venom and its fractions were used against 3rd-4th instar* Aedes aegypti* (27°C, 70% relative humidity, and 12 h of photoperiod). Assays were performed in quadruplicate using a solution of 1% ethanol as a negative control (solid line). (a) Crude venom. (b) Fraction P1. (c) Fraction P2. (d) Fraction P3.

The crude venom and P1, P2, and P3 fractions caused larval mortality that increased significantly with time (*F* = 101.1; *P* < 0.001, *F* = 384.3; *P* < 0.001; *F* = 242.0; *P* < 0.001 and *F* = 357.7; *P* < 0.001, resp.) and concentration (*F* = 205.0; *P* < 0.001; *F* = 1044; *P* < 0.001, *F* = 1952; *P* < 0.001 and *F* = 1188; *P* < 0.001, resp.). Crude venom and fractions effects on larval mortality differed significantly (*F* = 71.45; *P* < 0.001) from each other after 48 hours within the concentrations tested, except between P1 and P2. At 15 *μ*g/mL, more than 85% of larvae were dead after 96 hours in all assays (Figure 9), peaking at 99% for the fraction P3 (Figure 9(d)).

Various compounds from plants and animals can be listed as potential bioinsecticides as effective and safe alternatives to chemical insecticides [65, 66]. Venoms from spiders are a rich source of peptide insecticides, which were evolutionarily adjusted to achieve a broad range of receptors and ion channels in the nervous system of insects, possessing broad biotechnological potential [67–69].

Cesar et al. [16] described the structure of a new insecticidal compound named hydroxytrypargine isolated from the venom of* Parawixia bistriata*. The assay was performed injecting bees at a dose of 37 ng/g bee with a microsyringe, and an LD_50_ of 8 ± 2 ng/g was found for the *β*-carbonilic toxin and an LD_50_ of 29 ng/g for the crude venom. The present data corroborate the insecticidal activity of* P. bistriata* and also highlight the topic and/or oral effect of the crude venom and fractions of this spider species on* Aedes aegypti *with very low LC values for crude and even fractions of potential insecticides. Therefore, further characterization for the elucidation of the chemical nature of the venom components and a search for their targets will be addressed in the near future.

## 4. Conclusions

We observed the presence of biomolecules in* Parawixia bistriata *venom showing, individually or together, myotoxic, anticoagulant, edematogenic, mild genotoxic, insecticidal, and fungicidal activities.

Detailed analysis of bioactive proteins from the venom of* Parawixia bistriata* through more sequencing and biochemical-toxicological assays may result in better understanding of the pharmacological activity of this venom and the elucidation of its constituents, contributing to the advancement of toxinology and also to the direct or indirect development of products of medical or scientific interest.

## Supplementary Material

During the study of the venom of *P. bistriata* and functional characterization of some additional tests that have not integrated the main text of the article, such as the proteolytic activity on casein substrate and edema activity were performed. These results can be viewed in the supplementary material section.

## Figures and Tables

**Table 1:** Anticoagulant activity of *Parawixia bistriata* spider venom.

*Parawixia bistriata* (*μ*g)	Time of coagulation (min)
10	5:15 ± 0:23
25	7:45 ± 0:47^#^
50	10:35 ± 1:38^#^
100	22:25 ± 4:65^#^
200	Did not coagulate^∗∗#^
250	Did not coagulate^∗∗#^
CaCl_2_ (0.1 M)*	4:00 ± 0:26

*Inducing coagulation control. **After 48 hours of observation. The anticoagulant activity of the venom was observed by adding 10–250 *μ*g of *P*. *bistriata* crude venom into the plasma while monitoring clotting time. Mean values of the clotting time followed by the standard deviation expressed six individual experiments with triplicate samples. ^#^Significance level (*P* < 0.05) when compared to the control.

**Table 2:** Nuclear quantification of human lymphocytes treated with *Parawixia bistriata* spider venom.

Treatments (*μ*g/mL)	% cells/500 cells				CBPI ± S.D.
	^ a^Mono	^ b^Bi	^ c^Tri	^ d^Multi	
Control (+)					
6	48.5	38.7	7.6	5.2	1.643 ± 0.1
Control (−)					
—	50.3	39	7.3	3.4	1.604 ± 0.07
*P. bistriata *					
2.5	56.8	37.5	3.6	2.1	1.489 ± 0.05
5	50.4	40.2	5.4	4	1.590 ± 0.2
15	53.9	38.6	3.9	3.6	1.536 ± 0.09

^a^Mono: mononucleated, ^b^Bi: binucleated, ^c^Tri: trinucleated, ^d^Multi: multinucleated. Positive control (+): cisplatin (6 *μ*g/mL). Negative control (−): untreated cells in culture. CBPI: cytokinesis block proliferation index, that defines whether the cultures are multiplying normally after the addition of samples. The following formula was used according to Kirsch-Volders (1997) [6]: CBPI = 1 (mono) + 2 (bi) + 3 (tri + tetra)/500. Mean cells number followed by the standard deviation.

**Table 3:** Frequency of nucleoids with comet after treatment of human lymphocytes with *Parawixia bistriata* spider venom.

Treatments (*μ*g/mL)	Damaged nucleoids (%)	A.U.
^ a^Negative control		
—	22	22*
^ b^Positive control		
6	39.75	134.5^#^
*P. bistriata *		
2.5	38	49^#∗^
5	40.3	52.9^#∗^
7.5	57.1	76.9^#∗^
10	62.5	83.3^#∗^
15	70.6	97.7^#∗^
30	80.5	115.3^#∗^
60	83	117.1^#∗^
120	80.6	113.6^#∗^

^a^Negative control: culture of untreated cells, ^b^positive control: doxorubicin (6 *μ*g/mL). A.U.: arbitrary units (0–400) calculated according to [7]. ^#^Statistically different from the negative control (*P* < 0.05). *Statistically different from the positive control (*P* < 0.05).

**Table 4:** Lethal concentrations (LC) in *μ*g/mL of crude venom fractions from *Parawixia bistriata* for *Aedes aegypti* (Diptera: Culicidae) larvae.

Source	LC50	LC90
Crude venom	9	25
Fraction P1	6	17
Fraction P2	8	15
Fraction P3	7	16
