# Supplementary material for: Biochemical and Functional Characterization of Parawixia bistriata Spider Venom with Potential Proteolytic and Larvicidal Activities
Source: Biomed Res Int. 2014 May 7;2014:950538. doi: 10.1155/2014/950538 (PMC4033418; doi:10.1155/2014/950538)
Supplement: Supplementary file 1 — During the study of the venom of P. bistriata and functional characterization of some additional tests that have not integrated the main text of the article, such as the proteolytic activity on casein substrate and edema activity were performed. These results can be viewed in the supplementary material section. [file 950538.f1.doc]

**Biochemical and functional characterization of *Parawixia bistriata* spider venom with potential proteolytic and larvicidal activities**

Gizeli S. Gimenez, Antônio Coutinho Neto, Anderson M. Kayano, Rodrigo Simões-Silva, Frances T. T. Trindade, Alexandre A. Silva, Silvana Marcussi, Saulo L. da Silva, Carla F. C. Fernandes, Juliana P. Zuliani, Leonardo A. Calderon, Andreimar M. Soares^*^, Rodrigo G. Stábeli

**SUPPLEMENTARY MATERIAL**

Confirming the presence of proteolytic activity, the crude venom of *P. bistriata* was also active on the substrate casein in a directly proportional but non-linear, concentration-dependent manner.


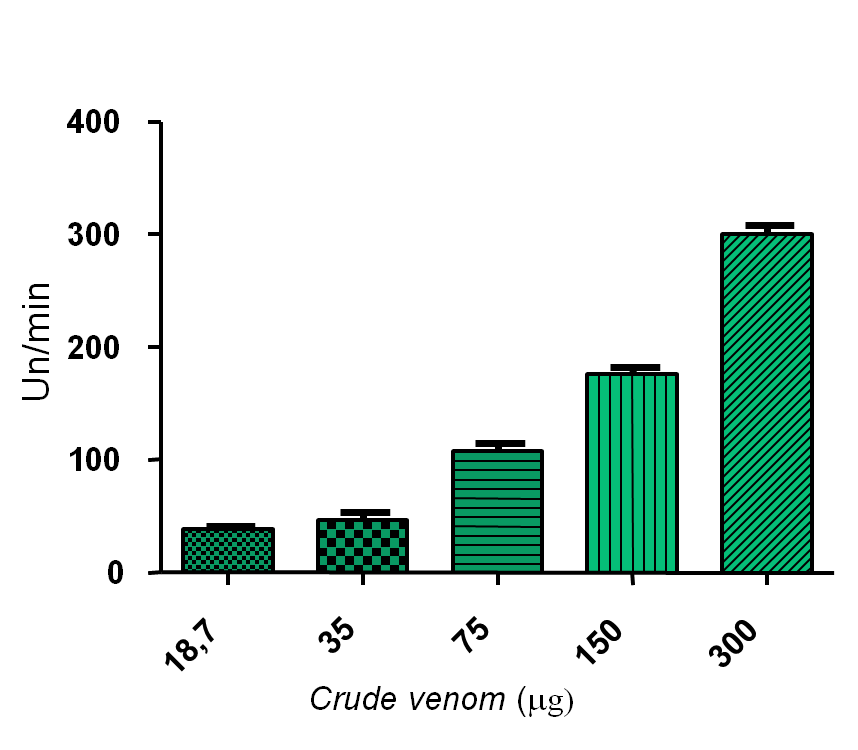


**Figure A. Caseinolytic activity of *Parawixia bistriata* venom.** 18.7 to 300 µg of crude venom was incubated in a solution of 1% casein for 30 minutes at 37 °C. One unit of protease activity was defined as the amount of enzyme capable of producing an increase in absorbance of 0.001 units/minute (Un/min) at 280 nm.

Regarding the edematogenic capacity, the crude venom showed dose-dependent activity at doses higher than 10 μg/animal. It was noted that the dose of 10 μg/animal was able to increase edema by 80% compared to edema caused by the negative control PBS after 30 minutes. For most of the tested doses, maximum edema occurred after 60 minutes with relative edema decay after 180 minutes.

It should be stated that higher doses of 100, 150 and 200 µg / animal were also tested for induction of edema, but no edematogenic activity significantly greater than that obtained with the dose of 50μg/animal was observed (data not shown).


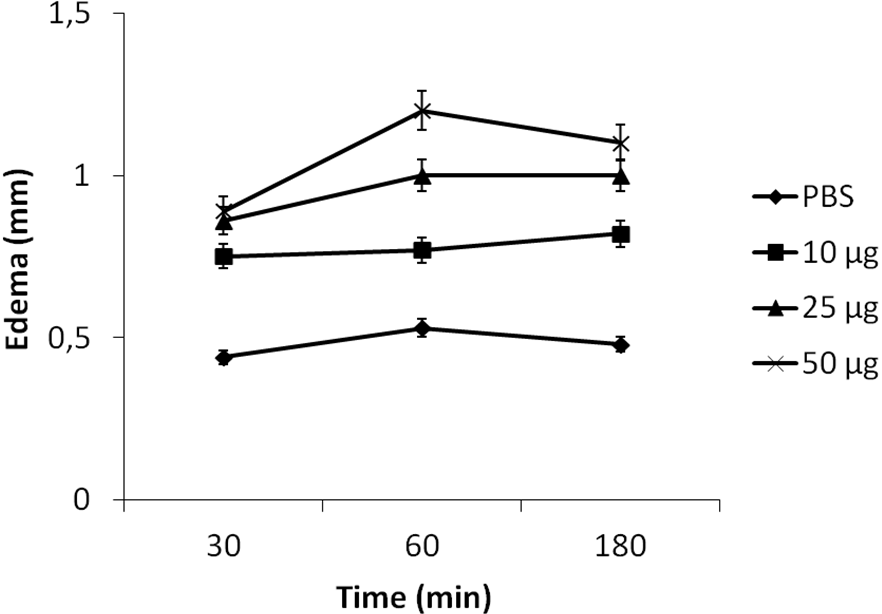


**Figure B. Edematogenic activity of *Parawixia bistriata venom.*** Paw edema was observed in mice injected in the subplantar region after injections of 10-200 µg/animal of *P. bistriata* crude venom diluted in 50 µL of PBS. PBS: negative control. The measurement of edema was performed using a low pressure spring gauge (Mitutoyo, Japan) at times of 30, 60 and 180 minutes.
